# Etiological Agents and Predisposing Factors of Superficial Fungal Infections in Northeastern Argentina

**DOI:** 10.3390/jof11040245

**Published:** 2025-03-23

**Authors:** Ana Clara Almada, Beda Mereles-Rodríguez, Miriam Chade, Isabel Belinchón-Romero, Enrique-Jorge Deschutter, José-Manuel Ramos-Rincón

**Affiliations:** 1 Department of Microbiology, Faculty of Exact, Chemical and Natural Sciences, National University of Misiones, Posadas N3304, Argentina; anaclara.almada@fceqyn.unam.edu.ar (A.C.A.); jorgedeschu@hotmail.com (E.-J.D.); 2 Mycology Section, High Complexity Laboratory of Misiones, Dr. Ramón Madariaga Acute Care Hospital, Posadas N3300, Argentina; bedamereles@fceqyn.unam.edu.ar (B.M.-R.); miriamchade@gmail.com (M.C.); 3 Department of Clinical Medicine, Miguel Hernández University of Elche, 03550 Alicante, Spain; ibelinchon@umh.es; 4 Department of Dermatology, Dr. Balmis General University Hospital-ISABIAL, 03010 Alicante, Spain; 5 Public Health and Communicable Diseases, National University of Misiones, Posadas N3304, Argentina; 6 Department of Internal Medicine, Dr. Balmis General University Hospital-ISABIAL, 03010 Alicante, Spain

**Keywords:** dermatomycoses, tinea, Argentina, hygiene, epidemiology

## Abstract

Superficial fungal infections (SFIs) account for approximately 2% of outpatient visits. Individual, environmental, and socioeconomic factors may increase susceptibility. The objective was to identify the etiological agents of SFIs and the predisposing factors. A cross-sectional descriptive study was conducted on patients attending the Level I Hospital in Puerto Piray, Misiones Province, Argentina, during a community intervention between 2022 and 2023. Statistical analysis was performed on the studied variables and their association with the identified etiological agents. A total of 138 clinical samples were collected from 107 patients with lesions suggestive of SFIs. Of the samples studied, 37% had an identified etiological agent. The majority were women (65%), those aged over 45 years (29%), and patients with underlying conditions (50%). A lack of access to sewage systems (100%) and potable water (19%) and overcrowding (19%) were observed. Dermatophytes (46%) were the most frequent etiological agents, with *Trichophyton tonsurans* being the primary species. An association was found between dermatophytosis and male sex (odds ratio [OR]: 4.4), ages 1–14 years (OR: 8.04), and overcrowding (OR: 5.38). In conclusion, the prevalence of SFIs was high in the studied population. The main etiological agents involved were anthropophilic dermatophyte fungi. Socioenvironmental conditions may contribute to the incidence of these pathologies.

## 1. Introduction

In 2019, skin and subcutaneous diseases had an incidence of 4.8 billion worldwide, with fungal skin diseases bearing the highest burden. It is estimated that between one and two billion people globally suffer from superficial fungal infections (SFIs), and the World Health Organization (WHO) considers their prevalence to be around 20–25%. The main etiological agents are dermatophytes and yeast-like fungi of the genera *Candida* spp. and *Malassezia* spp. [1,2,3,4].

The presence of individual, environmental, and socioeconomic factors can increase the host’s susceptibility to SFIs. Other clinical findings have been associated with these conditions, such as corticosteroid therapy, keratinization disorders, and other hereditary factors [5,6].

In Argentina, there is limited knowledge due to the lack of unified national records on the incidence, prevalence, and distribution of fungal diseases in the population. Data from a multicenter study conducted in Argentina indicated that 47% of fungal isolates found in clinical settings were responsible for dermatomycosis. Among the most frequently observed agents reported were dermatophytes (68.7%), yeast species (16.9%), and *Malassezia* spp. (12.9%) [7]. In the Province of Cordoba, a cross-sectional study conducted in 2015 in public and private establishments reported the isolation of species of the genus *Trichophyton* spp. (33%) as the main agent causing dermatomycosis, followed by *Candida* spp. (17.8%) [8]. There have been few mycological studies in Misiones Province, with *Microsporum canis* being the main agent of SFIs, recorded in children under 10 years of age [9]. In Paraguay, adjacent to Misiones Province, the prevalence of SFIs in glabrous skin observed by Mesa-Aquino in 2019 was 38% (*n* = 899). The identified agents were dermatophytes (56%), *Malassezia* spp. (42%), and *Candida* spp. (2%). Among the dermatophytes, the most frequent were *Trichophyton rubrum* (70%) and *M. canis* (17.3%) [10].

Based on the research conducted in the area and the lack of clinical and epidemiological data, a community intervention study was designed to identify people with suspected SFIs in the municipality of Puerto Piray in Misiones Province, Argentina (Northeast Argentina).

The objective of this work was to determine the etiological agents of SFIs, evaluate the sensitivity of direct mycological examination, and analyze the predisposing factors of SFIs in patients attending a Level I Hospital after being identified during a community intervention.

## 2. Materials and Methods

### 2.1. Study Design, Context, and Population

A cross-sectional study was conducted in the locality of Puerto Piray (GPS: 26°28′02″ S 54°43′05″ W; −26.4672678, −54.7180771) (Misiones Province, Argentina) from December 2022 to November 2023. The municipality covers an area of 354 km^2^ and is in the Montecarlo Department, 201 km away from the capital of Misiones, in the Alto Paraná region. It has 9985 inhabitants distributed across 34 neighborhoods. The main economic activity revolves around the timber industry.

The recruitment of the minimum number of participants was calculated based on the estimation of the effect size for a difference of 20–30%, a power of 80%, and a significance level of 95%, requiring at least 88 patients.

The study population included all individuals who presented with lesions compatible with SFIs and who agreed to participate by signing informed consent forms. The identification and recruitment of beneficiaries were based on participatory research strategies within the community from the mycology service intervention, from November 2022 to December 2023. The characteristics of the community intervention are included in Appendix A.

In the selection of subjects, the presence of suspected lesions on the skin, scalp, and nails was evaluated. In the case of patients under 13 years of age, their parents or guardians provided consent for sample collection without prejudice to the necessary information and participation of the child who gave their approval.

### 2.2. Study Variables

Age, sex, duration of the lesions prior to the evaluation, anatomical site of the lesion, underlying disease, use of medications prior to the evaluation, level of education, physical activity, and socioenvironmental factors related to access to the sewage system and potable water network, the presence of functional bathrooms in the home, the number of rooms, and the number of cohabitants (to calculate overcrowding) were recorded. The description of the collected variables is detailed in Appendix B. Some lesions found in the study participants is in Appendix C.

### 2.3. Mycological Procedure and Processing

**Figure 1:**
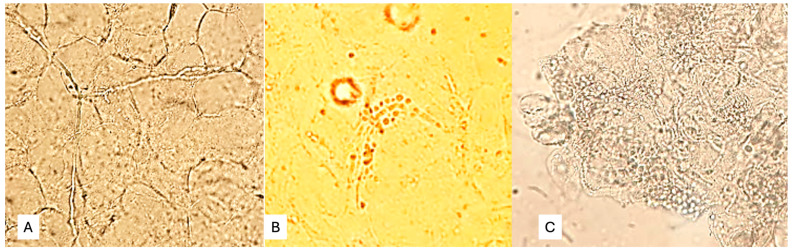
(**A**) Fungal structures in mycelial form (pseudohyphae), (**B**) yeasts with or without pseudomycelium, (**C**) Yeasts in clusters characteristic of *Malassezia* spp.

Skin scale, scalp, and nail samples were processed for direct microscopic examination (DME) in the laboratory of the Puerto Piray Hospital. The samples were collected in sterile Petri dishes. The DME of skin scales, hair, and nail debris was performed with 20% potassium hydroxide, with which a preliminary or definitive report was prepared as appropriate. The applied DME enabled the detection of fungal structures in mycelial form (pseudohyphae, Figure 1A), yeasts with or without pseudomycelium (Figure 1B), or yeasts in clusters characteristic of *Malassezia* spp. (Figure 1C).

The samples were sent for inoculation in specific media, culture analysis, and identification of fungal species according to classical mycological methodology to the Dra. Martha Medvedeff Mycology Laboratory of the Faculty of Exact, Chemical, and Natural Sciences of the National University of Misiones, Extension Project “Medically Relevant Fungal Isolates,” Posadas, Misiones, Argentina. For each genus of *Candida* spp., *Malassezia* spp., and dermatophytes, species were identified according to classical mycological taxonomy classification tests. Cultivation of fungi was performed on Sabouraud dextrose agar, potato dextrose agar, and selective agar. In cases of *Malassezia* spp., the diagnostic procedure involved the clinical findings and DME, where yeast-like structures in clusters and short hyphae were observed. Differentiation of *Candida* species was achieved using CHROMagarTM Candida (CHROMagar, Saint-Denis, France) along with examination of the morphological characteristics of the yeasts (formation of hyphae, blastoconidia, chlamydoconidia, or arthroconidia) using microculture on Tween agar. Dermatophytes were identified based on their macroscopic and microscopic morphological characteristics [11].

### 2.4. Statistical Analysis 

Data analysis was performed using the statistical software R 4.4.1., R Studio 2024.04.2+764.pro1, and Microsoft Excel. The results were expressed as the prevalence rate of SFIs in the study population, frequencies of the predisposing factors and etiological agents, and the means with standard deviations (SD). The sensitivity, specificity, and positive and negative likelihood ratios of DME were determined [12]. The analyses of the identified exposure factors were compared with the detected etiological agents, determining the degree of association for each using chi-squared or Fisher’s tests as appropriate. The strength of this association was expressed as the odds ratio (OR). Additionally, an adjusted logistic regression model was carried out, using as independent variables the predisposing factors that showed a *p*-value < 0.05.

### 2.5. Ethical Aspects 

This study adhered to the guidelines set forth by the Declaration of Helsinki (1964) and the provisions of the Guide for Research on Human Subjects, Resolution 1480/11. The work complies with the parameters for approval by the Research Ethics Committee of Misiones Province. All the patients signed informed consent forms before participating in the study, and for minors, the consent was signed by the parents or guardians.

## 3. Results

### 3.1. Sociodemographic Characteristics 

A total of 138 clinical samples were collected from 107 patients with suspected SFI lesions. All the patients had been permanent residents of the municipality for at least two years prior to the study, and they carried out their work or educational activities regularly in areas close to their homes. Table 1 summarizes the epidemiological and clinical characteristics of the study patients.

### 3.2. Direct Microscopic Examination and Identification of Etiological Agents 

The prevalence of SFIs in the total number of patients (107) for the study period was 37% (95% CI: 0.28–0.47).

**Figure 2:**
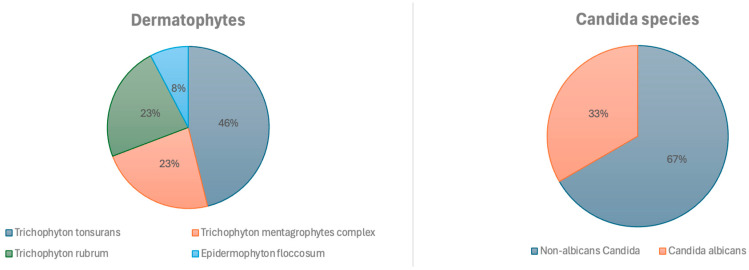
Identified fungi.

A total of 138 samples were analyzed, 48 of which were positive, 34% (95% CI: 0.27–0.43). Of the total samples, 25.4% (*n* = 35) were taken from glabrous skin, 24.6%—from toenails, 21.7% (*n* = 30)—from the skin of the hands, face, feet, or groin, 13.8% (*n* = 19)—from the scalp, and 10.9% (*n* = 15)—from fingernails. Figure 2 shows the results of the positive DME (+), which identified filamentous fungal structures (FFS) or yeast-like fungal structures (YFS), and the pathogens were identified according to the sample collection site.

**Figure 3:**
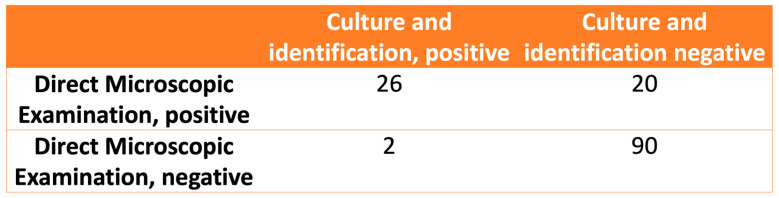
The relationship between direct microscopic examination and fungal culture and identification.

The relationship between the observations in the DME and fungal identification is shown in Figure 3. The calculated values for the sensitivity, specificity, positive likelihood ratio, and negative likelihood ratio of the DME were 92.8% (CI 95%: 75.0–98.7%), 81.8% (CI 95%: 73.1–88.3%), 5.1 (CI 95%: 3.4–7.7), and 0.08 (CI 95%: 0.02–0.33), respectively.

### 3.3. Characteristics of Patients with SFIs

The main characteristics studied in the 48 patients with positive DME or culture, according to sex, are detailed in yeast-like fungal structures (YFS) (Table 2).

### 3.4. Predisposing Factors for Superficial Fungal Infections

Table 3 shows the characteristics studied as predisposing factors for superficial fungal infections. In the bivariate analysis, a higher risk of SFIs was highlighted in men and in people with a lower level of education, and a lower risk was highlighted in those with higher education. However, in the multivariate analysis using a logistic regression model, none of these variables was significant.

### 3.5. Analysis of the Predisposing Factors According to Fungal Etiology

A bivariate analysis was conducted to determine the statistical association of each of the probable predisposing factors found with diagnosed candidiasis, dermatophytosis, and *Malassezia* infection.

A statistically significant association was found between sex, age group, and overcrowding and dermatophytosis. However, when adjusting through the logistic regression method, the independent variables did not show statistical significance. For *Malassezia* spp., an association was found with the 15–24 age group (Table 4).

## 4. Discussion

This study was the first conducted on SFIs in a population from Alto Paraná, Misiones (Northeast Argentina). It involved 107 patients with suspected SFI lesions who attended consultations and provided clinical samples.

During the study period, a prevalence of 37% of SFIs was recorded among patients who attended the hospital in Puerto Piray, with dermatophytes being the main isolates and *T. tonsurans* being the predominant species. This is interesting because, compared to what was reported by Rustan et al. [8], a frequency of 65.2% of dermatomycoses was found in public and private centers in the city of Córdoba (central region of Argentina), with the most prevalent agents being dermatophytes of the genus *Trichophyton* spp. However, in peri-urban populations where access to health services is scarce, studies are infrequent; González et al. [13] identified various species of fungi causing SFIs in a community in the province of Córdoba (Argentina), with *T. rubrum* being the most frequent species, followed by yeasts of the genus *Candida* spp., *C. albicans* and *C. tropicalis.*

Similar results to those observed in the present study were found in Colombia by Estrada-Salazar and Chacón Cardona [5], where a frequency of 35% (*n* = 146) was identified, and in Paraguay by Meza-Aquino et al. [10], with a prevalence of SFIs in glabrous skin of 38% (*n* = 899). However, when identifying the most frequently involved dermatophyte, Silva-Rocha in Brazil [14], similarly to Meza-Aquino, found *T. rubrum* to be the most common [10]. In Venezuela, Capote et al. [15] also reported a frequency of 34% in samples processed for the diagnosis of some type of SFIs. This contrasts with the findings of Bitew in his 2017–2018 cross-sectional study at the Advanced Medical Laboratory Arsho (Addis Ababa, Ethiopia), where fungi were detected and/or isolated in 67.98% (*n* = 318) of the patients with suspected SFI lesions [16]. The differences observed in our work compared to different geographical regions could be explained by various factors such as climate, urban environment, socioeconomic level, and cultural habits that may contribute to the appearance of dermatomycoses. In their work, Uhrlab et al. [17] concluded that there was a sudden epidemiological shift from *T. rubrum* to *T. indotineae*, which generated an epidemic in countries such as India, the United Arab Emirates, Oman, and Iran and is now spreading to Europe, becoming a public health problem due to the number of affected people and the conditions of poverty where it occurs.

It could be speculated that epidemiological patterns are also changing in Argentina, where outbreak reports or epidemiological alerts have involved species that are uncommon for the studied population, such as *T. benhamiae*, a zoophilic species whose ecological niche is guinea pigs (Maldonado et al. [18]), and cases of *T. tonsurans* as agents of tinea capitis in adolescents attending barbershops in the city of Buenos Aires [19].

*Malassezia* infection appeared in our study as an agent causing pityriasis versicolor, pityriasis/*Malassezia* folliculitis, and seborrheic dermatitis, where it was the second most frequently isolated etiological group, with a statistical association with the age group of 15–24 years (*p*-value < 0.05), with the probability of suffering from this SFI being 5 times higher in this group. In line with this, Meza et al. [10] reported similar results, where *Malassezia* infection was the second most common dermal condition, occurring more frequently in those aged 16–30 years, with the most common clinical expressions (91.6%) being hyperchromic and hypochromic lesions on the face, neck, back, and chest. On the other hand, similar findings were observed by Henshaw et al. [20] in Nigeria, where 79% of adolescents aged 13–19 years suffered from pityriasis versicolor, making it the most common infectious dermatosis in this population. However, this work differs from the findings of Ortiz-Florez et al. [21] in Colombia, where the median age was 33 years, and men were affected more frequently.

Although candidiasis was the least frequent lesion observed in our study, it mainly manifested in the fingernails and, in one case, in the submammary skin. It is noteworthy that *C. albicans* mainly affected housewives, while *C. parapsilosis* was isolated in the nails of patients with psoriasis. Our work is consistent with the findings published by Relloso et al. [22], where yeast-like fungi were predominant in the fingernails. In a study conducted by Zisova et al. [23] in patients with psoriasis, out of the 228 cases examined, 62% had onychomycosis, with the main etiological agents being dermatophytes and yeast infections of the genus *Candida* spp. This study linked the presence of dystrophic nails in patients with psoriasis to a higher predisposition to SFIs. Additionally, according to Elsner et al. [24], the prevalence of *Candida* spp. was significantly higher in the psoriatic patients compared to the control subjects (*p* ≤ 0.001). This group of patients should be closely monitored for symptoms of candidiasis during treatment with IL-17 inhibitors. Furthermore, as highlighted in the literature review by Armstrong, onycholysis is a common finding in patients with psoriasis or those exposed to skin maceration due to excess moisture, increasing the risk of developing comorbid opportunistic infections. This is because the compromised nail bed creates a favorable environment for fungal growth [24].

Regarding the activities of the study participants, housewives, students, and administrative employees were the most frequent occupations. However, when analyzing patients with SFIs, we observed that while housewives and cleaning staff were the predominant occupations among women, men were mainly engaged in industrial and transportation activities, with a higher statistical association in men (*p*-value of 0.014). This partially coincides with the findings of Jiménez-Olvera et al. [25] in Mexico, where patients affected by SFIs were more frequently engaged in household chores, followed by employees, merchants, students, field workers, and drivers. The higher activity of affected men in the industrial sector is also reflected by Villavicencio-Soledispa et al. [26], who studied 265 workers aged 20 to 52 years, of whom 69% developed dermal conditions; this is similar to the results obtained by Aveiga Maldonado et al. [27], where 45.2% were from the labor sector, 35.7% worked in household administration, and 7.1% were students.

The analysis revealed that 50% of the affected individuals had an underlying disease, mainly cardiovascular pathologies and diabetes mellitus. This finding is consistent with the study conducted by Eftekhari et al. [28] during the 2022–2023 period, where a significant association was found between diabetes and onychomycosis. The prevalence rate of onychomycosis in diabetic patients was almost double that in non-diabetic patients (17.9% versus 9.7%; *p* < 0.001). Additionally, in this same study, hypertension was observed as a comorbidity in these patients, although no statistically significant association was found. Jiménez-Olvera also noted the relationship between diabetes and foot SFIs. Another study, conducted by Agrawal et al. [29], evaluated 102 patients with onychomycosis and diabetes, where the most affected population was men over 60 years old.

The duration of the lesions was examined, and 50% of the analyzed population had been affected for more than a year. However, when evaluating cases associated with SFIs, it was observed that the duration increased to 2 years in 50% of the cases, and 33.3% had used topical medications to treat the lesions, mainly mixtures of steroids, antibiotics, and antifungals. These findings suggest the chronicity of the lesions, possibly due to the inappropriate use of topical medication or the absence of clinical suspicion for their management. This first observation is consistent with the case–control study conducted by Jha et al. [30], which determined that the application of topical corticosteroids is a risk factor for recurrent dermatophytosis, and with the study of Thakran et al. [31], who found that the duration of superficial dermatophytosis varied from 3 to 14 months in patients treated with steroids, a shorter time than that estimated in the present work. Similarly, Uhrlab et al. [17] stated that the use of broad-spectrum topical treatments could be related to the persistence of lesions and that the existence of a recalcitrant *Trichophyton* spp. epidemic in India suggests that the use of this medication would contribute to this situation. On the other hand, Đorđević Betetto [32] described a case of tinea incognita in a patient with psoriasis, where he stated that widespread dermatophyte infections tend to be neglected and can present concomitantly with other dermatoses, complicating their diagnosis.

Quality of life, overcrowding, and hygiene conditions are directly associated with a higher prevalence of infectious diseases. Additionally, a higher educational level is associated with more financial resources to invest in health, while a lower income level or unemployment is associated with stress. The results of this study and many other investigations on the same subject point out that socioenvironmental and sanitary hygiene conditions significantly affect the prevalence of dermatological diseases, including fungal infections. The most important factors that influence the development of infections are the limitations in access to the sewer network and the supply of drinking water [6]. Additionally, living conditions, such as overcrowding, and low education were more frequent predisposing variables in patients with SFIs [5]. According to the evaluation of DME as a diagnostic test for SFIs, the calculated sensitivity value was very high (92.8%); it is recommended as a cheap, rapid, and easy-to-use test for the diagnosis of SFIs. However, DME has advantages as a prompt diagnostic method, but also disadvantages, such as the need for expert knowledge and the inability to identify the etiological agent.

## 5. Conclusions

In this study, we determined that the SFI prevalence was very high (37%) in a population group with unfavorable socioenvironmental conditions, such as overcrowding, low education level, and residence in regions with underdeveloped sewage and water supply systems. Women in the 45–65 age group were the most frequently infected. The etiological agents most frequently isolated from the processed clinical samples were dermatophytes, followed by *Malassezia* spp. and *Candida* spp. A statistical analysis determined that dermatophytosis was more prevalent among the male patients and in the patients living in overcrowded conditions, while *Malassezia* infection was more common among the patients aged 15–24 years.

Although this work has limitations, the results showed an epidemiological overview of SFIs in our region and can serve as the basis for establishing additional future research as well as effective strategies of education and measures to prevent the spread of infections.

## Figures and Tables

**Table 1:** Sociodemographic characteristics of the 107 study patients.

	N	%
Sex		
Female	82	76.6
Male	25	23.4
Age: mean (SD), range	42 (20), 1–82	
Comorbidities		
Any comorbidity	31	29.0
Cardiovascular diseases	14	13.1
Diabetes mellitus	7	6.5
Immune disorders	6	5.6
Topical treatment *	36	33.6
Duration > 1 year	53	49.5
Occupation		
Housewives	28	26.2
Students	19	17.7
Administrative and service employees	13	12.1
Retired	10	9.3
Industry and transport employees	9	8.4
Teachers	8	7.5
Healthcare workers	8	7.5
Others and unemployed	12	13.3
Higher education	19	17.7
Physical activity	40	37.4
Environment/home		
No access to the sewage system	107	100
No access to potable water	22	20.6
Overcrowding	18	16.8
Absence of a bathroom in the home	4	3.7

* Prior to mycological diagnosis and includes antibiotics, antifungals, or corticosteroids, or combinations thereof.

**Table 2:** Characteristics studied in patients with superficial fungal infections (positive DME and/or culture) by sex.

	Total N (%)	Male N (%)	Female N (%)
Age groups (years)			
1–14	5 (10.4)	4 (8.3)	1 (2.1)
15–24	7 (14.6)	3 (6.2)	4 (8.4)
25–44	9 (18.7)	3 (6.2)	6 (12.5)
45–65	16 (33.3)	2 (4.2)	14 (29.1)
>65	11 (23)	5 (10.5)	6 (12.5)
Underlying disease			
Diabetes mellitus	7 (14.6)	3 (6.2)	4 (8.4)
Cardiovascular	9 (18.7)	4 (8.3)	5 (10.4)
Autoimmune	5 (10.4)	2 (4.2)	3 (6.2)
Oncological	1 (2.1)	1 (2.1)	0 (0)
Other	2 (4.2)	0 (0)	2 (4.2)
No underlying disease	24 (50)	15 (31.3)	9 (18.7)
Education level			
No education	2 (4.2)	2 (4.2)	0 (0)
Primary	28 (58.3)	11 (22.9)	17 (35.4)
Secondary	15 (31.1)	4 (8.4)	11 (22.9)
Higher	3 (6.2)	0 (0)	3 (6.2)
Occupation			
Housewife	12 (25)	0 (0)	12 (25)
Student	11 (22.9)	5 (10.4)	6 (12.5)
Retired	8 (16.7)	4 (8.4)	4 (8.4)
Cleaning staff	4 (8.4)	0 (0)	4 (8.4)
Transport/industry staff	5 (10.4)	5 (10.4)	0 (0)
Other	8 (16.7)	3 (6.2)	5 (10.4)
Socioenvironmental factors			
Latrine	2 (4.2)	1 (2.1)	1 (2.1)
No sewage system	48 (100)	17 (35.4)	31 (64.6)
No access to potable water	9 (18.7)	6 (12.4)	3 (6.2)
Overcrowding	9 (18.7)	6 (12.4)	3 (6.2)

Note: N: number; %: percentage of the total.

**Table 3:** Risk factors for superficial fungal infections.

	Superficial Fungal Infections (*n* = 48) N (%)	No Superficial Fungal Infections (*n* = 90) N (%)	OR	95% CI	*p*-Value
Sex					
Men	17 (35.4)	16 (17.8)	2.5	1.04–6.09	0.03 *
Women	31 (64.6)	74 (82.2)	0.4	0.16–0.95	0.03
Age groups					
1–14	5 (10.4)	5 (5.6)	1.9	0.42–9.05	0.31
15–24	7 (14.6)	7 (7.8)	2.0	0.56–7.23	0.24
25–44	9 (18.8)	31 (34.4)	0.4	1.16–1.08	0.07
45–65	16 (33.3)	35 (38.9)	0.8	0.35–1.73	0.58
>65	11 (22.9)	12 (13.3)	1.9	0.69–5.27	0.16
Underlying disease					
Any underlying disease	24 (50)	30 (33.3)	-	-	-
Diabetes mellitus	7 (14.6)	8 (8.9)	1.7	0.5–5.93	0.39
Cardiovascular	9 (18.8)	13 (14.4)	1.3	0.47–3.8	0.62
Autoimmune	5 (10.4)	8 (8.9)	1.2	0.28–4.42	0.76
Oncological	1 (2.1)	1 (1.1)	-	-	-
Other	2 (4.2)	0 (0)	-	-	-
No underlying disease	24 (50)	60 (66.7)	-	-	-
Education Level					
No education	2 (4.2)	2 (2.2)	1.9	0.13–27.01	0.61
Primary	28 (58.3)	30 (33.3)	2.7	1.27–6.15	0.006 **
Secondary	15 (31.3)	39 (43.3)	0.6	0.26–0.31	0.2
Higher	3 (6.2)	19 (21.2)	0.2	0.045–0.92	0.027 ***
Occupation					
Housewife	12 (25)	25 (27.8)	0.8	0.35–2.05	0.84
Student	11 (22.9)	12 (13.3)	1.3	0.42–3.8	0.61
Retired	8 (16.7)	5 (5.6)	3.3	0.90–13.94	0.06
Cleaning staff	4 (8.3)	3 (3.4)	2.6	0.42–18.64	0.23
Transport/industry staff	5 (10.4)	19 (21.2)	0.9	0.36–2.49	1
Other	8 (16.7)	6 (6.7)	-	-	-
Socioenvironmental					
Latrine	2 (4.2)	2 (2.2)	1.9	0.13–27.05	0.61
No sewage system	48 (100)	90 (100)	-	-	-
No access to potable water	9 (18.8)	13 (14.4)	2.1	0.66–6.39	0.18
Overcrowding	9 (18.8)	9 (10)	2.1	0.66–6.39	0.18

Note: bold is reserved for *p* < 0.01; N: number; %: percentage of the total. * 0.01; *p* ≤ 0.05, ** 0.001; *p* ≤ 0.01, *** *p* ≤ 0.001.

**Table 4:** Predisposing factors associated with diagnosed superficial fungal infections.

	Dermatophytosis (*n* = 13)		Malassezia Infection (*n* = 9)		Candidiasis (*n* = 6)	
	N	OR (95% CI)	N	OR (95% CI)	N	OR (95% CI)
Sex						
Men	7	4.4 (1.4–14.3) *	3	1.6 (0.38–6.99)	1	0.6 (0.07–5.54)
Women	6	0.2 (0.07–0.7) **	6	0.6 (0.14–2.57)	5	1.6 (0.18–14.2)
Age groups						
1–14	4	8.8 (2.09–37.02) ***	0	NC	0	NC
15–24	1	0.7 (0.08–5.97)	3	5.4 (1.17–24.46) ******	0	NC
25–44	3	0.7 (0.18–2.74)	3	1.2 (0.29–5.23)	1	0.5 (0.05–4.21)
45–65	4	0.7 (0.21–2.52)	2	0.5 (0.09–2.33)	3	1.7 (0.33–9.01)
>65	1	0.39 (0.04–3.15)	1	0.6 (0.07–5.11)	2	2.6 (0.45–15.3)
Underlying disease						
Diabetes mellitus	1	0.6 (0.79–5.47)	1	1.3 (0.11–8.82)	1	1.7 (0.18–15.4)
Cardiovascular	0	NC	3	2.9 (0.66–12.57)	0	NC
Autoimmune	1	0.8 (0.09–6.57)	1	1.2 (0.14–10.58)	2	5.5 (0.90–33.5)
Oncological	1	10.2 (0.60–17.4)	0	NC	0	NC
Other	0	NC	0	NC	0	NC
No underlying disease	10	2.3 (0.60–8.76)	4	0.5 (0.12–1.91)	3	0.6 (0.12–3.24)
Education level						
No education	2	11.2 (1.43–87.27)	0	NC	0	NC
Primary	6	1.2 (0.38–3.78)	5	1.8 (0.45–6.98)	5	7.4 (0.85–65.60)
Secondary	5	0.9 (0.29–3.13)	3	0.7 (0.18–3.19)	1	0.3 (0.03–2.62)
Higher	0	NC	1	0.6 (0.07–5.41)	0	NC
Occupation						
Housewife	2	0.5 (0.09–2.21)	2	0.7 (0.15–3.87)	3	2.8 (0.55–14.90)
Student	3	1.6 (0.39–6.23)	2	1.5 (0.28–7.57)	0	NC
Retired	1	0.8 (0.09–6.57)	1	1.2 (0.14–10.6)	2	5.5 (0.9–33.47)
Cleaning staff	2	4.4 (0.75–25.16)	0	NC	0	NC
Transport/industry staff	4	2.3 (0.65–8.31)	2	1.2 (0.24–6.39)	1	0.8 (0.09–7.60)
Other	1	-	2	-	0	-
Socioenvironmental						
Latrine	1	3.4 (0.32–35.16)	0	NC		NC
No access to potable water	6	5.8 (1.74–19.58) ****	2	1.5 (0.30–8.04)	1	1.0 (0.11–9.51)
Overcrowding	5	5.4 (1.53–18.9) *****	2	0.8 (0.09–7.00)	2	3.6 (0.60–21.40)

Note: NC, not calculated (variables with values of 0 cannot have an odds ratio calculated). Significant factors for dermatophytosis: * male sex (7/13), *p*-value: 0.014; ** female sex (6/13), *p*-value: 0.014; *** age group of 1–14 years (4/13), *p*-value: 0.008; **** no access to potable water (6/13), *p*-value: 0.007; ***** overcrowding (5/13), *p*-value: 0.014. Significant factors for *Malassezia* infection: ****** age group of 15–24 years (3/9), *p*-value: 0.048.

**Figure A1:**
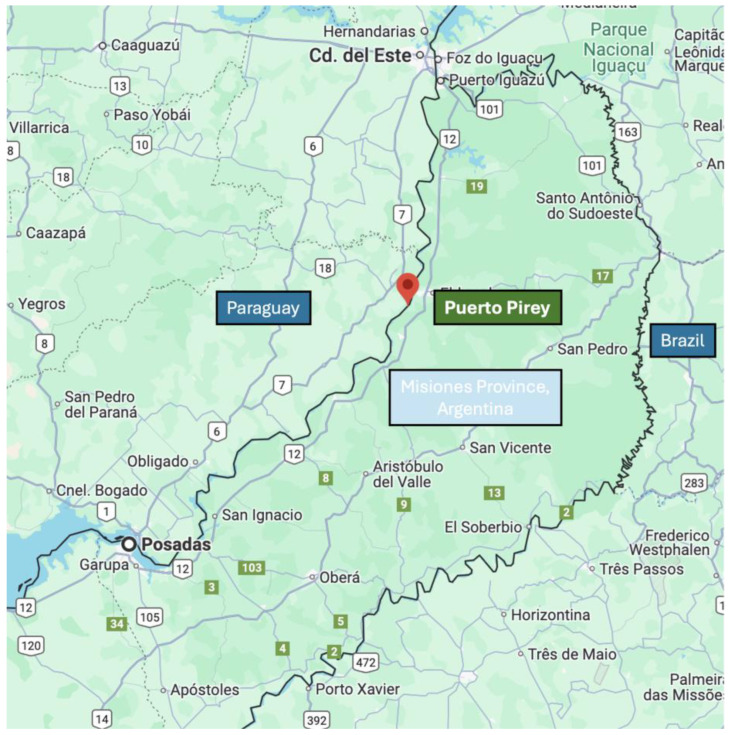
Satellite view of Misiones Province between Paraguay and Brazil, Google Maps, 2024, maps.google.com.

**Figure A2:**
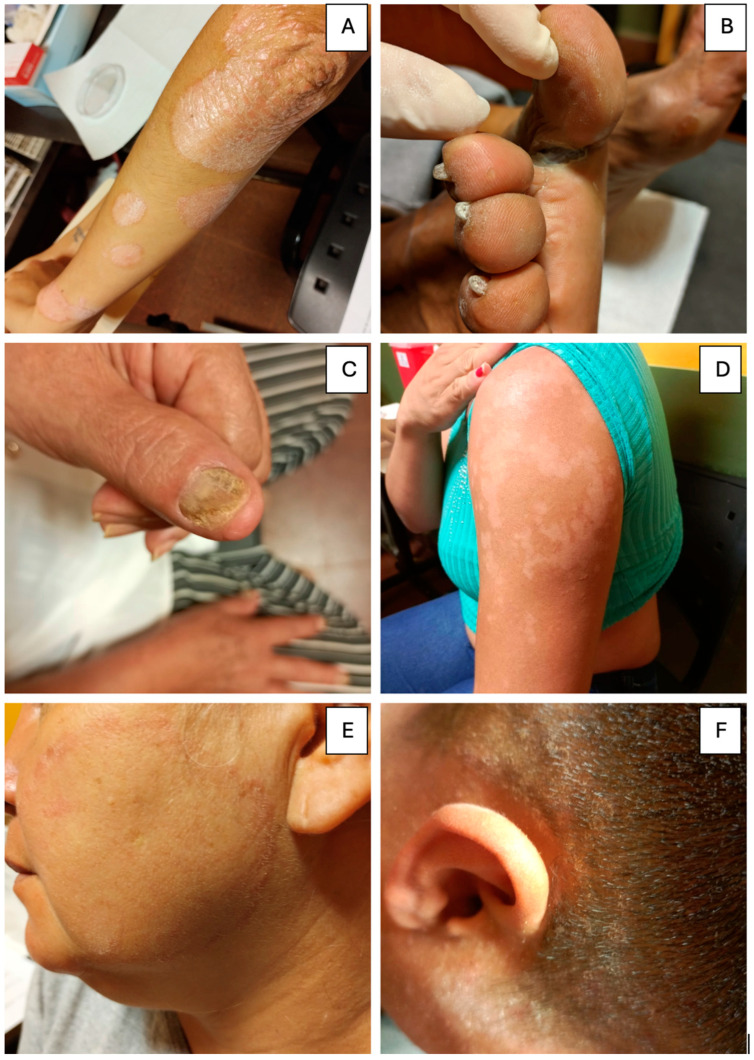
Lesions found in the study participants. (**A**) Non-mycotic plaque lesions in a female patient. (**B**) Interdigital and nail lesion caused by a dermatophyte. (**C**) Onychodystrophy and paronychia caused by the *Candida albicans* complex. (**D**) Extensive hypochromic lesions on the arm caused by *Malassezia* spp. (**E**) A lesion with an erythematous border caused by *T. tonsurans*. (**F**) A scaly lesion on the scalp.

## Data Availability

The data that support the findings of this study are available upon request from the corresponding author. The data are not publicly available due to privacy or ethical restrictions.

## Abbreviations

The following abbreviations are used in this manuscript:

DME	Direct microscopic examination
FFS	Filamentous fungal structures
YFS	Yeast-like fungal structures
OR	Odds ratio
95% CI	Confidence intervals of 95%

## Appendix A. Community Intervention

Community interventions are of great importance to reaching the affected population and act as facilitators in the diagnostic process, while also reinforcing health promotion and disease prevention activities in the territories, strengthening the management capacities of health policy actors with responsibilities at the primary care level and encouraging the active search and follow-up by the health team, as proposed by the National Community Health Program (PNSC), Ministerial Resolution 844/2022.

The work was carried out in the locality of Puerto Piray, Misiones Province (Figure A1). The municipality covers an area of 354 km² and is in the Montecarlo Department, 201 km away from the capital of Misiones, in the Alto Paraná region. It has 9985 inhabitants, according to the 2010 National Census of Population, Households, and Housing, distributed across 34 neighborhoods.

The identification and recruitment of beneficiaries were based on participatory research strategies in the community through the intervention of the mycology service, from November 2022 to December 2023. Work was conducted in conjunction with the members of the health team of the mentioned hospital, accessing local media, municipal public institutions, and cultural and educational centers through the development of open talks to the community. Awareness on the subject and training of health personnel were carried out (Extension Project Res. CD No. 300/23, FCEQyN-UNaM).

## Appendix B. Collected Variables

The following variables were analyzed to determine the predisposing factors for SFIs:

Age (in years): a quantitative variable operationalized in strata, with five categories: 0–14 years, 15–24 years, 25–44 years, 45–64 years, and >65 years.

Sex: dichotomous variable, male, female.

Duration of lesions (in years): prior to the time of patient evaluation; a quantitative variable starting from 1 (corresponding to ≤1 year).

Anatomical site of the lesion: a polytomous variable, categorized as facial skin, glabrous skin (excluding hands, feet, groin, and face), inguinal skin, hand skin, foot skin, scalp, fingernails, and toenails.

Underlying disease: a polytomous variable, grouped by cardiovascular diseases, diabetes mellitus (DM), immune system diseases, oncological diseases, and other diseases.

Occupation: a polytomous variable, grouped into housewives, retirees, students, teachers, cleaning staff, transport and industry staff, and others.

Use of medications prior to the evaluation: a dichotomous variable (yes or no) depending on whether topical treatment with antifungals–corticosteroids–antibiotics was used.

Level of education: a polytomous variable, categorized as no education, primary, secondary, and higher education.

Socioenvironmental factors: a set of variables integrated for analysis, each of a dichotomous nature, categorized as latrines (presence–absence), access to potable water (presence–absence), sewage treatment system (presence–absence), and overcrowding (yes–no).

DME result of clinical samples: a dichotomous variable, positive–negative.

Isolated and identified etiological agents: a polytomous variable, categorized into the genera of *Candida* spp., *Malassezia* spp., and dermatophytes. For each genus, species were identified according to classical mycological taxonomy classification tests.
